# Laryngeal squamous cell carcinoma: a functional “two-hit” model of papillomavirus–Epstein–Barr virus synergistic carcinogenesis

**DOI:** 10.3389/fcimb.2026.1768893

**Published:** 2026-02-20

**Authors:** Fang Wang, Hui Huangfu

**Affiliations:** 1 Department of Otolaryngology Head and Neck Surgery, The First Hospital of Shanxi Medical University, Taiyuan, Shanxi, China; 2 The First Clinical Medical School of Shanxi Medical University, Taiyuan, China

**Keywords:** EBV, EMT, functional “two-hit” mode, HPV, immune evasion, laryngeal squamous cell carcinoma, Warburg effect

## Abstract

Smoking, alcohol consumption, and high-risk human papillomavirus (HPV) infection are recognized risk factors for laryngeal squamous cell carcinoma (LSCC); however, approximately 20%–30% of patients lack typical exposure histories. Furthermore, HPV-positive patients exhibit significant prognostic heterogeneity, suggesting the complexity of multifactorial synergistic carcinogenesis. Recent studies have indicated that the unique anatomy and microenvironment of the larynx may mediate synergistic interactions between pathogens such as Epstein–Barr virus (EBV) and HPV. This functional “two-hit” model drives metabolic reprogramming, disrupts immune responses, and promotes immune evasion, thereby potentially fueling the malignant progression of LSCC. However, coexistence patterns and dynamic interaction mechanisms remain unclear. This systematic review of field advances aims to elucidate potential synergistic mechanisms between HPV and EBV, providing theoretical foundations for pathogen-based multitarget diagnostics and clinical treatment strategies to advance the precision prevention and treatment of LSCC.

## 1 Introduction

Laryngeal cancer, a common malignant tumor of the head and neck, is predominantly laryngeal squamous cell carcinoma (LSCC), accounting for 90%–95% of cases (Bray et al., 2024). Despite continuous medical advances, the overall prognosis of patients with laryngeal cancer has improved only marginally over the past decades, underscoring the urgent need for a deeper understanding of its pathogenesis (Gatta et al., 2015). Smoking and alcohol consumption are recognized as the major risk factors for LSCC development. However, the etiology of laryngeal cancer is highly heterogeneous, with approximately 20%–30% of patients lacking these typical exposure histories, suggesting the potential involvement of other significant carcinogenic factors (Huang et al., 2023).

Among these, infection with high-risk genotypes of human papillomavirus (HPV), particularly HPV16, has been firmly implicated as an independent oncogenic factor in LSCC (Chow, 2020). However, the clinical course of HPV-associated LSCC exhibits considerable heterogeneity. Patient responses to treatment and ultimate outcomes vary widely, indicating that HPV alone is insufficient to fully explain the pathogenesis and prognostic spectrum of the disease (Wicinski et al., 2018; Panuganti et al., 2021; Zhang et al., 2025). This observed heterogeneity strongly suggests that cooperative interactions between HPV and additional cofactors are pivotal in modulating carcinogenesis and clinical behavior.

In this context, Epstein–Barr virus (EBV), a ubiquitous herpesvirus with well-documented oncogenic potential, has also been detected in a reproducible subset of LSCC tumors (Schindele et al., 2022; de Lima et al., 2021). Notably, the concurrent detection of HPV and EBV in LSCC tissue samples has been reported across multiple cohorts, with prevalence rates varying from 1.3% to as high as 15% in virologically defined subgroups; this variation is largely attributable to methodological and geographic differences (Drop et al., 2017; Vazquez-Guillen et al., 2018; Schindele et al., 2022). Critically, beyond mere co-detection, this dual infection status is consistently associated with distinct clinicopathological features, most notably a higher degree of tumor differentiation, affirming its status as a non-random biological event with potential mechanistic significance (Gupta et al., 2020b).

Nevertheless, the precise patterns of coexistence, temporal dynamics, and comprehensive molecular mechanisms through which HPV and EBV functionally synergize to fuel LSCC progression remain largely unresolved. To address this gap, we propose a functional “two-hit” model. This “two-hit” framework outlines a plausible biological sequence: HPV may deliver the first hit by profoundly reprogramming the epithelial niche and disrupting host differentiation, which in turn may facilitate the establishment of EBV latency. Subsequent collaborative cross talk between viral proteins from both pathogens could amplify impairments across key cellular hallmarks, collectively lowering the potential threshold for malignant transformation and possibly accelerating tumor progression in HPV–EBV-coinfected laryngeal epithelium. In the following sections, we systematically synthesize the evidence for this putative synergy in regulating metabolic reprogramming, suppressing DNA damage repair, inducing epithelial–mesenchymal transition, and enacting multilayered immune evasion. By integrating virological, metabolic, and immunological perspectives, we aim to provide a unified mechanistic framework that may clarify a distinct pathway of LSCC development and lay a theoretical foundation for future translational strategies targeting this coinfected subset.

## 2 Potential synergistic carcinogenic mechanism of HPV–EBV based on a functional “two-hit” model

### 2.1 A proposed model: HPV–EBV coinfection synergistically promotes epithelial malignancy

A comprehensive understanding of the putative synergy between human papillomavirus (HPV) and Epstein–Barr virus (EBV) in laryngeal squamous cell carcinoma (LSCC) necessitates an integrated perspective on their distinct yet complementary oncogenic paradigms, which converge to form a collaborative “two-hit” model. The basis of this model rests on the independent pathogenic mechanisms of each virus. High-risk HPVs, most notably HPV16, promote carcinogenesis via their early oncoproteins E6 and E7 (Gupta et al., 2018; Chow, 2020).

The E6 protein inactivates the key tumor-suppressor p53 through ubiquitin-mediated degradation, impairing the DNA damage response and apoptotic pathways, while E7 targets the retinoblastoma protein (pRb) for degradation. This liberation of E2F transcription factors thereby drives unchecked cell cycle progression (Yang et al., 2019; Zhang et al., 2025). Beyond overriding cell cycle checkpoints, a pivotal role of E6/E7 lies in the profound disruption of the host epithelial differentiation program—a process central to both the viral life cycle and the cross talk with EBV (Nawandar et al., 2015).

EBV, in turn, utilizes a distinct oncogenic toolkit. It infects epithelial cells via receptors such as Ephrin type-A receptor 2 (EPHA2), a receptor tyrosine kinase that transduces pro-tumorigenic signals upon viral engagement (Locard-Paulet et al., 2016; Bu et al., 2022). Following infection, EBV can establish latency, during which proteins such as latent membrane protein 1 (LMP1) act as constitutively active signaling molecules. These activate pathways, including NF-κB, to enhance cell survival and proliferation (Cao et al., 2021). The switch from latency to lytic replication is tightly linked to terminal epithelial differentiation and is directly modulated by host factors, such as Krüppel-like factor 4 (KLF4) (Nawandar et al., 2015).

The proposed synergistic model posits that HPV infection acts as the initial priming hit. By expressing E6 and E7, HPV establishes a differentiation-defective microenvironment in the basal epithelial layer. This altered cellular state directly interferes with EBV’s differentiation-dependent lytic replication, favoring a shift toward persistent latency—characterized by the sustained expression of latent oncoproteins such as LMP1 (Makielski et al., 2016; Guidry et al., 2019). This HPV-mediated reprogramming creates a permissive niche for EBV persistence, constituting the first hit.

Within this preconditioned microenvironment, the second hit unfolds through functional cross talk between persistent HPV oncoproteins and latent EBV factors, thereby amplifying oncogenic effects. For instance, co-expression of HPV E6 and EBV LMP1 synergistically compromises DNA damage response pathways while hyperactivating pro-survival signals such as NF-κB, thereby exacerbating genomic instability and apoptosis resistance (Shimabuku et al., 2014). Furthermore, EBV entry via EPHA2 in coinfected cells may provide an additional layer of early synergy: engagement of the receptor by viral glycoproteins aberrantly activates its intrinsic pro-invasive and pro-angiogenic signaling, potentially accelerating the early stages of malignant progression (Chatzikalil et al., 2024; Lee et al., 2024).

**Figure 1:**
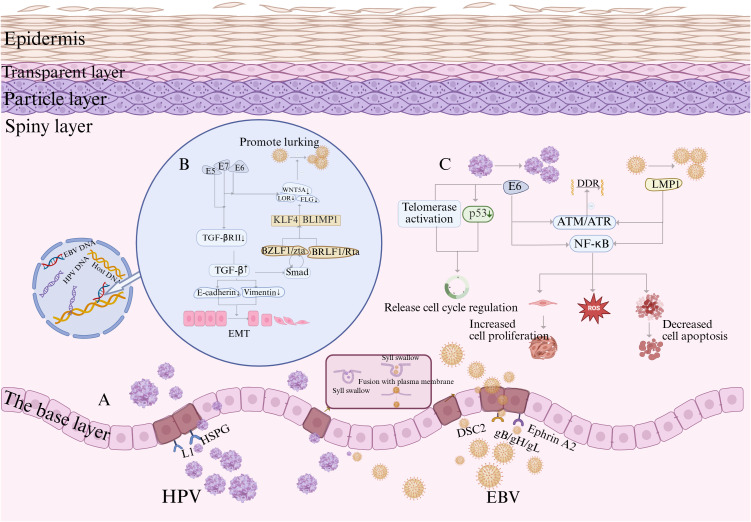
Proposed mechanisms of HPV and EBV coinfection and their hypothesized collaborative role in oncogenesis. **(A)** Key steps in HPV and EBV infection of epithelial cells. HPV binds to cell surface heparan sulfate proteoglycan (HSPG) molecules via its L1 protein and subsequently enters cells via endocytosis. EBV enters cells via endocytosis, followed by membrane fusion mediated by the viral glycoproteins gH/gL and gB, to complete the infection. The host protein EphA2 acts as a crucial cofactor in this process, interacting with EBV glycoproteins to promote viral internalization and fusion. **(B)** The diagram illustrates how EBV and HPV differentially exploit the host TGF-β/Smad pathway to promote survival and pathogenicity. EBV uses this pathway to activate the lytic replication switch BZLF1/Zta and establishes a Zta-TGF-β-positive feedback loop to amplify the lytic effect. Conversely, HPV suppresses the upstream receptors of this pathway via the E5/E7 proteins to evade its growth-inhibitory effects. Ultimately, both strategies cause TGF-β accumulation, jointly promoting tumor progression processes, including EMT. **(C)** When HPV E6 and EBV LMP1 are co-expressed, they inhibit the ATM/ATR signaling axis, a core component of DDR. Concurrently, they activate the NF-κB pathway, which promotes malignant progression by enhancing proliferation, inhibiting apoptosis, and increasing reactive oxygen species (ROS) production. Additionally, HPV E6 disrupts cell cycle checkpoint control by degrading p53 and activating telomerase. Created using BioRender.com.

In summary, this “two-hit” framework outlines a plausible biological sequence: HPV delivers the first hit by profoundly reprogramming the epithelial niche and disrupting host differentiation, which in turn facilitates the establishment of EBV latency. Subsequent collaborative cross talk between viral proteins from both pathogens amplifies impairments across key cellular hallmarks, collectively reducing the threshold for malignant transformation and accelerating tumor progression in HPV–EBV-coinfected laryngeal epithelium (**Figure 1A**).

### 2.2 A functional model: coinfection synergistically suppresses DDR to drive malignant transformation

HPV and EBV coinfection synergistically disrupts genomic stability, and both viruses induce basal DNA damage through viral replication. The coexistence of low-risk HPV (LR-HPV) and EBV triggers limited malignant transformations. Although it increases secondary DNA damage and impairs DNA damage response (DDR) efficiency, leading to somatic mutation accumulation and precancerous lesions, it remains insufficient to drive complete malignant transformation (Uehara et al., 2021). Conversely, co-expression of HR-HPV and EBV is hypothesized to form a potent carcinogenic axis (**Figure 1C**). When the E6 oncoprotein of HR-HPV is co-expressed with EBV latent membrane protein LMP-1, it significantly suppresses core DDR pathway factors, such as the Ataxia Telangiectasia Mutated and ATM- and Rad3-Related Signaling Axis (ATM/ATR) signaling axis, while activating the NF-κB pathway (Shimabuku et al., 2014). Sustained activation of the NF-κB signaling pathway, primarily mediated by LMP1, promotes cell proliferation, increases resistance to apoptosis, and disrupts the reactive oxygen species (ROS) homeostasis, conferring survival advantages to cancer cells (Xin et al., 2022; Khenchouche et al., 2023). However, LMP1 activation alone is necessary but insufficient for coinfection-driven carcinogenesis. The synergistic action of the HPV E6 protein via mechanisms, including p53 degradation and telomerase activation, can bypass cell cycle checkpoint regulation, establishing sufficient and necessary conditions for malignant transformation. Once established, this transformed state may no longer depend on the initial peak NF-κB activity level (experiments confirmed its subsequent decline), suggesting that E6 and LMP1 may synergistically reprogram cells to achieve transformation autonomy. In animal models (e.g., mouse embryonic fibroblasts), co-expression of E6 and LMP1 significantly enhances tumorigenesis, validating their cascading carcinogenic efficacy in suppressing DDR and hijacking signaling pathways (Shimabuku et al., 2014).

### 2.3 Model of coinfection: maintaining viral latency to promote persistent infection and EMT progression

EBV sustains infection through a dual latent lytic life cycle. During latency, the virus persists long-term in B cells or epithelial cells as inclusions with restricted viral gene expression. During the lytic cycle, it undergoes three phases: immediate-early (transactivation of BZLF1/Zta and BRLF1/Rta), early (expression of viral replication proteins), and late (assembly of structural proteins, such as capsid antigens and membrane proteins), culminating in infectious virions. EBV lytic activation is highly dependent on epithelial differentiation progression, directly driven by the host transcription factors KLF4 and BLIMP1, triggering BZLF1/BRLF1 promoters (Nawandar et al., 2015). However, HPV significantly disrupts this process via its oncoproteins, E6/E7–HPV16. E6/E7 blocks epithelial differentiation by downregulating KLF4 target genes, including *FLG*, *LOR*, and *WNT5A*, with E7 specifically delaying early differentiation marker expression, thereby undermining the differentiation signals required for EBV lytic replication (Guidry et al., 2019). Thus, the precise regulation of KLF4 by HPV oncoproteins constitutes a fundamental aspect of the microenvironmental reprogramming model. High-risk HPVs fine-tune KLF4 through multilayered mechanisms: E7 upregulates KLF4 protein levels by suppressing miR-145, while E6 may alter KLF4 activity and stability by interfering with its posttranslational modifications (Gunasekharan et al., 2016). Furthermore, within the context of early coinfection in the basal layer, as described in Section 2.1 and occurring prior to terminal differentiation during active E6/E7-mediated reprogramming, this regulatory process contributes to establishing a cellular environment permissive for EBV lytic gene expression. Collectively, within the differentiation-deficient microenvironment established by HPV, the paucity of host differentiation cues favors a shift of EBV from a lytic replication mode to a latent state. We hypothesize that this shift is not merely a default outcome but a critical adaptive strategy. It ensures viral persistence in an otherwise non-permissive cellular context and, more importantly, establishes the essential foundation for the subsequent immune evasion and sustained oncogenic synergy between latent EBV proteins (e.g., LMP1) and persistent HPV oncoproteins. Furthermore, HPV–EBV coinfection directly increases genomic instability. Integration of HR-HPV genes into the host genome is a key event in HPV-driven carcinogenesis, a process that is potentially amplified by the coexistence of EBV. Studies indicate that the presence of EBV may increase the rate of HPV16 integration sevenfold (odds ratio [OR], 7.11; 95% confidence interval [CI]: 1.70–29.67) (Szostek et al., 2009). Another study found that EBV infection increased the likelihood of HPV16 genomic integration into the host genome fivefold (OR, 5.00; 95% CI: 1.15–21.80) (Kahla et al., 2012).

The transforming growth factor beta (TGF-β) signaling pathway may serve as a core hub for EBV and HPV cross-regulation of viral replication and transformation potential. The TGF-β1/Smad pathway is a critical switch in the EBV lytic cycle. It induces latent EBV reactivation by activating BZLF1/Zta expression, while Zta itself can promote TGF-β secretion via Smad-dependent or Smad-independent feedback mechanisms, forming a positive feedback loop that amplifies EBV lytic reactivation (Liang et al., 2002; Iempridee et al., 2011). However, this pathway has a dual effect on cancer progression. TGF-β, a core inducer of epithelial–mesenchymal transition (EMT), drives cancer cell migration and invasion by downregulating E-cadherin and upregulating vimentin (Velapasamy et al., 2018), directly promoting metastasis. Conversely, HPV disrupts TGF-β signaling via its early oncogenes, E5 and E7, synergistically downregulating TGF-βRII expression and blocking TGF-β/Smad signaling. This interference is thought to promote early carcinogenesis and could synergize with EBV to potentially accelerate EMT progression (**Figure 1B**) (French et al., 2013).

### 2.4 Proposed metabolic synergy: coinfection enhances the Warburg effect to drive malignancy

**Figure 2:**
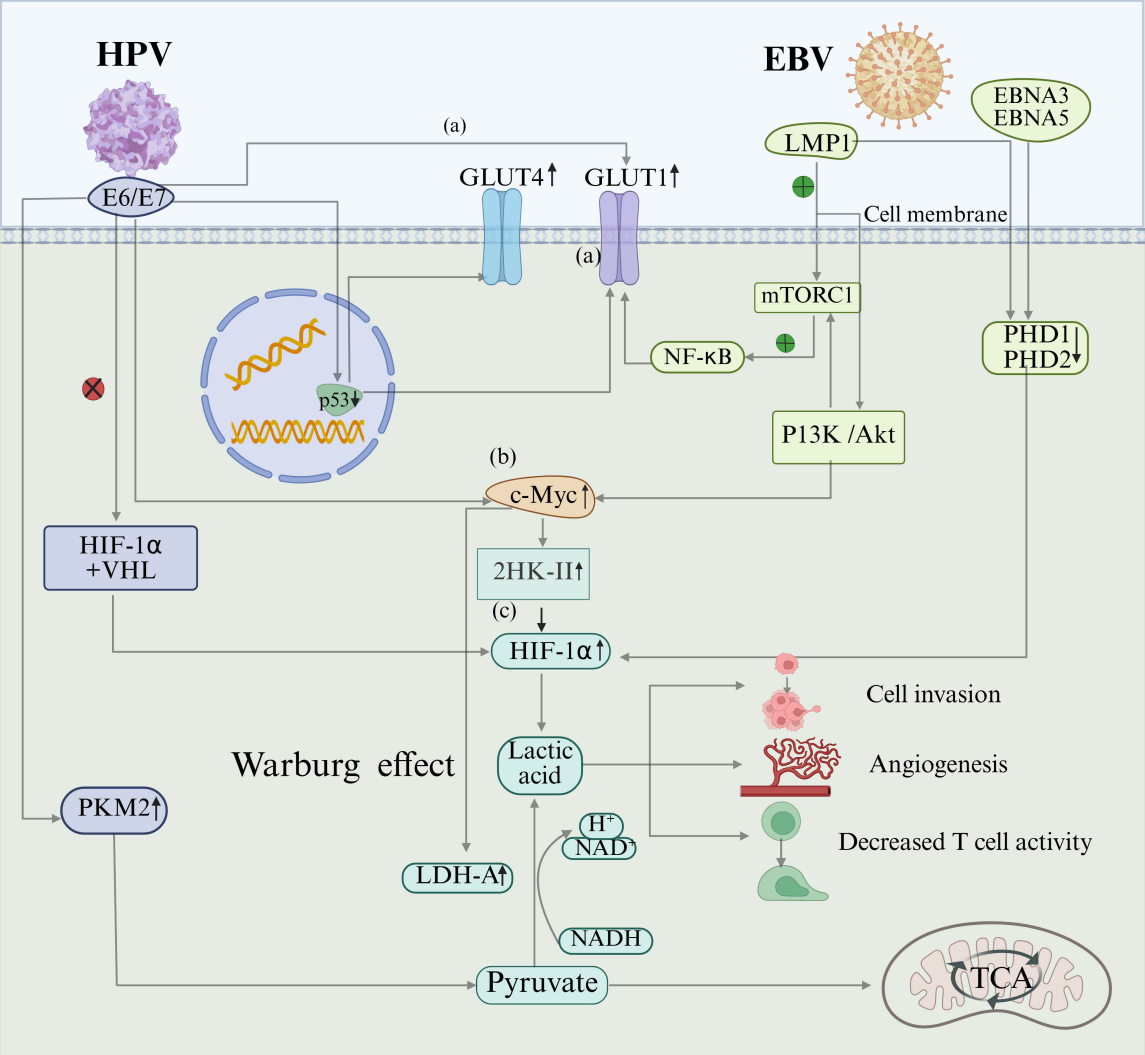
A model of HPV and EBV synergy in potentially inducing the Warburg Effect. Molecular models of HPV and EBV synergistically induce aerobic glycolysis (Warburg effect) in host cells via multiple signaling pathways. **(A)** GLUT upregulation: HPV E6 directly activates GLUT1. E7 upregulates GLUT1/4 via both p53-dependent and -independent pathways. EBV LMP1 induces GLUT1 expression by activating the mTORC1-NF-κB axis. **(B)** HK-II activation: HPV E6/E7 elevates the c-Myc level. EBV LMP1 stabilizes c-Myc protein via the PI3K/Akt-GSK3β-FBW7 pathway, jointly promoting HK-II expression and glycolytic activation. **(C)** Stabilization of hypoxia-inducible factor HIF-1α: HPV E6/E7 blocks HIF-1α binding to the VHL protein. EBV inactivates PHD1/2 via EBNA5/EBNA3, respectively, inhibiting HIF-1α ubiquitination and degradation, thereby leading to its accumulation. The synergistic effects of these mechanisms induce lactate accumulation, creating an acidic tumor microenvironment that promotes tumor cell invasion, angiogenesis, and suppression of T-cell function. Created using BioRender.com.

Metabolic reprogramming accompanying cancer progression centers on the Warburg effect (aerobic glycolysis). Aerobic glycolysis is the process by which tumor cells preferentially convert glucose into pyruvate via glycolysis, and subsequently into lactate, even under oxygen-sufficient conditions, bypassing the efficient energy production of mitochondrial oxidative phosphorylation (Paul et al., 2022). This process confers proliferative advantages to cancer cells by enhancing glycolytic flux and lactate accumulation while shaping an immunosuppressive tumor microenvironment (Heawchaiyaphum et al., 2023). HPV and EBV, as key oncogenic viruses, synergistically disrupt host metabolic programs through their encoded oncoproteins (**Figure 2**). At the glucose uptake level, E6 and E7 proteins of HPV directly activate glucose transporter 1 (GLUT1) expression, degrading the tumor suppressor p53 to release the transcriptional repression of GLUT1 and GLUT4 (Ancey et al., 2018), thereby upregulating GLUT1 via these dual mechanisms. However, LMP1 of EBV activates the mammalian target of rapamycin signaling complex 1-nuclear factor kappa-light-chain enhancer of activated B cells (mTORC1-NF-κB) signaling axis, inducing GLUT1 gene transcription and promoting its membrane localization, significantly enhancing glycolytic flux (Zhang et al., 2017). At the level of glycolytic activation, HPV relies on E6/E7 to elevate c-Myc levels, directly upregulating the glycolytic rate-limiting enzyme hexokinase II (HK-II) (Flier et al., 1987). LMP1 of EBV stabilizes c-Myc via the Phosphoinositide 3-Kinase (PI3-K)/Akt-Glycogen Synthase Kinase 3 Beta (GSK3β)-F-box and WD repeat domain-containing 7 (FBW7) signaling axis pathway, driving HK-II overexpression (the c-Myc-HK-II axis is essential for EBV metabolic reprogramming) while upregulating all glycolytic enzymes (Xiao et al., 2014). Finally, at the metabolic center regulatory level, HPV 16 oncoproteins E6/E7 block the interaction between hypoxia-inducible factor-1α (HIF-1α) and the E3 ubiquitin ligase von Hippel–Lindau (VHL) tumor-suppressor protein, inhibiting ubiquitin-mediated degradation (Knuth et al., 2017). However, EBV disrupts the HIF-1α hydroxylation-dependent degradation pathway via EBNA5 inactivation of prolyl hydroxylase-domain protein 1 (PHD1) and EBNA3 inhibition of prolyl hydroxylase-domain protein 1 (PHD2) (Darekar et al., 2012), with both viruses collectively elevating HIF-1α levels. Additionally, LMP1 of EBV stabilizes Siah 1 protein in human epithelial cells, inducing PHD degradation and preventing VHL-HIF-1α interaction, elevating HIF-1α levels in EBV-infected human cancer cell lines (Kondo et al., 2006). These mechanisms cause the abnormal accumulation of HIF-1α under normoxic conditions. HIF-1α, a master regulator of metabolism, drives angiogenesis, cell proliferation, and invasion by activating glycolytic pathways.

Aerobic glycolysis hyperactivates LDH-A, accelerating the conversion of pyruvate to lactate even under oxygen-sufficient conditions. Lactate accumulation triggers multiple vicious cycles. (1) Lactate acidification activates matrix metalloproteinases (MMPs), promoting tumor invasion (Hirschhaeuser et al., 2011; Cardeal et al., 2012). (2) Immunosuppression inhibits T-cell/dendritic cell function and synergizes with viral proteins to facilitate immune evasion (Fischer et al., 2007). (3) Angiogenesis synergistically promotes neovascularization via the HIF-1α/vascular endothelial growth factor (VEGF) axis (EBV-dominant) and direct lactate induction by VEGF/TGF-β (HPV-dominant) (Fischer et al., 2007; Polet and Feron, 2013). (4) Enhanced migration: lactate and EBV-LMP1 promote cell migration (Dawson et al., 2008; Liu et al., 2012).

### 2.5 A mechanistic framework: coinfection synergistically promotes immune evasion

Immune surveillance is a well-established characteristic of tumor cells (Hanahan and Weinberg, 2011). At the innate immune level, general virology studies show that HPV–EBV jointly suppresses the Toll-like receptor (TLR) pathways to facilitate immune evasion. TLRs are specialized pattern recognition receptors that play a crucial role in initiating antiviral immune responses (Rakoff-Nahoum and Medzhitov, 2009). HPV E6/E7 proteins significantly downregulate TLR2/3/5/7/9 expression (Hasan et al., 2013; Cigno et al., 2020), whereas EBV targets and inhibits TLR2/9 function through lytic proteins (Zhang et al., 2024), such as BGLF5 and BALF1 (Reiser et al., 2011). Furthermore, HPV-mediated TLR9 downregulation may significantly impair the ability of the host to detect EBV, and the association between TLR9 downregulation and increased EBV infection risk further demonstrates the synergistic promotion of immune evasion by HPV–EBV coinfection (Jabłońska et al., 2020).

**Figure 3:**
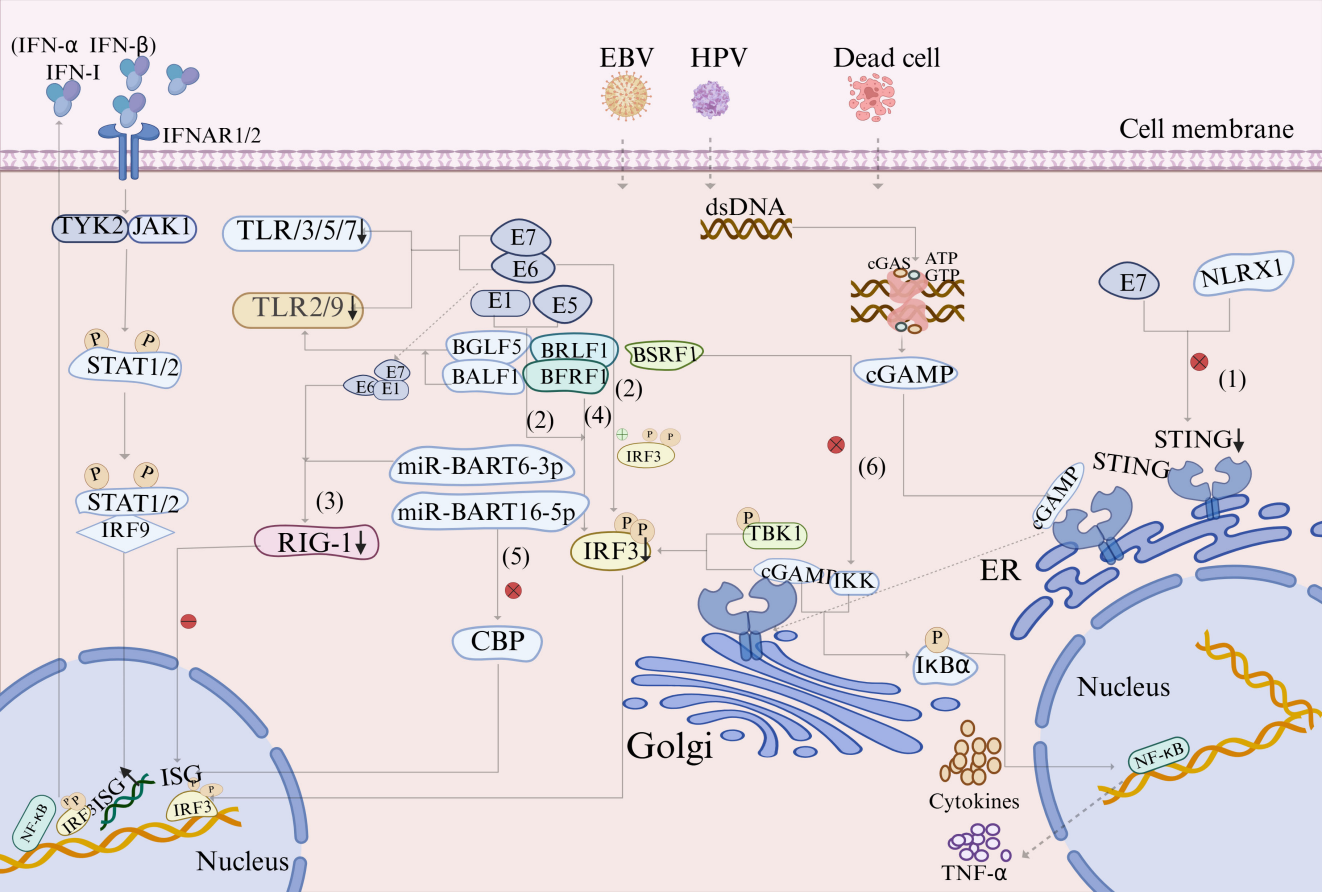
Proposed model: HPV and EBV coinfection synergistically targeting innate immune pathways to facilitate immune evasion.Molecular mechanism by which HPV and EBV coinfection promotes tumor immunosuppressive microenvironment formation by synergistic targeting and inhibition of innate immune signaling pathways, such as TLR and cGAS-STING. Downregulation of TLR pathways: When HPV and EBV coinfect, HPV16 E6/E7 proteins downregulate TLR2/3/5/7/9 expression, while EBV lytic proteins BGLF5 and BALF1 suppress TLR2/9 function. The cGAS-STING pathway, the core host cell mechanism for recognizing abnormal cytoplasmic dsDNA and inducing type I interferon (IFN-I production, is synergistically disrupted by viruses via multiple mechanisms, thereby promoting immune evasion. This pathway is initiated by cGAS sensing abnormal dsDNA, synthesizing the second messenger cGAMP, and subsequently activating the STING protein. Activated STING translocates to the Golgi apparatus, recruits TBK1 kinase, and phosphorylates and activates IRF-3 and NF-κB, ultimately inducing the expression of IFN-I and pro-inflammatory factors, including IL-6, TNFα, and IL-1β. The generated IFN-I binds to the IFNAR1/2 receptor, activates IRF9 via the JAK-STAT pathway, and promotes ISG transcription, establishing an antiviral state. However, HPV and EBV jointly suppress this pathway through the following mechanisms: (1) HPV16 E7 protein mediates STING degradation via its NLRX sequence, directly disrupting cGAS-STING signaling. (2) HPV16 E1/E5 proteins inhibit IRF3 activity, while the E6 protein directly binds and downregulates IRF3 expression. (3) HPV E1/E6/E7 proteins interfere with RIG-I expression, thereby reducing ISG production. (4) EBV-encoded proteins, including BFRF1, BRLF1, and BILF4, inhibit IRF3 activation. (5) EBV miR-BART16-5p degrades the transcriptional coactivator CBP, while miR-BART6-3p downregulates RIG-I expression. (6) BSRF1 inhibits IKK complex activation and IFN-I production. These multi-tiered inhibitory effects significantly weaken the innate immune response of the host, enabling the virus to successfully evade IFN-I-mediated antiviral effects. This further promotes immunosuppression and malignant progression within TME. Created using BioRender.com.

In the innate immune system that defends against pathogens, the cyclic GMP-AMP synthase (cGAS) stimulator of interferon genes (STING) signaling pathway can be activated by abnormal intracellular double-stranded DNA (dsDNA) signals to exert anti-inflammatory or pro-tumor effects (Tufail et al., 2025). Insights from viral immunology suggest that coinfection with HPV and EBV may amplify the pro-tumor activity of this pathway, aiding cancer cells in evading immune surveillance (**Figure 3**) (Zhang and Zhang, 2025). Specifically, E1 and E5 proteins of HPV16 suppress interferon regulatory factor 3 (IRF3) function, reducing type I interferon (IFN-1) production (Li et al., 2025; Scott et al., 2020). HPV E6 directly binds IRF3 to decrease IFN-α/β/γ/κ generation (Lee et al., 2001), while E1/E6/E7 broadly interferes with RIG-I expression, diminishing IFN-stimulated gene (ISG) production (Lee et al., 2001; Li et al., 2025). Recent studies have revealed that the NLRX1 sequence of HPV16 E7 degrades STING, disrupting the cGAS-STING pathway (Luo et al., 2020). EBV, via its capsid protein BRLF1, reduces IRF3/7 expression to inhibit IFN-β production (Bentz et al., 2010). The early protein BFRF1 suppresses IRF3 phosphorylation, dimerization, and nuclear translocation, thereby inhibiting interferon-β (IFN-β) generation (Wang et al., 2020). The envelope protein LF2 (BILF4) blocks IRF3 activation, reduces IFN-α and IFN-β production (Wang et al., 2009; Wu et al., 2009), and uses miR-BART16-5p to degrade the ISG co-activator CREB-binding protein (CBP), thereby downregulating IFN-α (Hooykaas et al., 2017). The miR-BART6-3p targets the RIG-1 receptor to reduce IFN-β production (Lu et al., 2017). The EBV membrane protein BSRF 1 inhibits I-κB kinase (IKK) complex activation by blocking IKKα/IKKβ heterodimer formation, thereby interfering with IFN-β production and promoting viral replication (Chen et al., 2025). Coinfection with these two viruses may synergistically target the core IRF3 pathway and downstream ISGs, jointly establishing an IFN-suppressed immune environment that promotes tumor cell growth.

**Figure 4:**
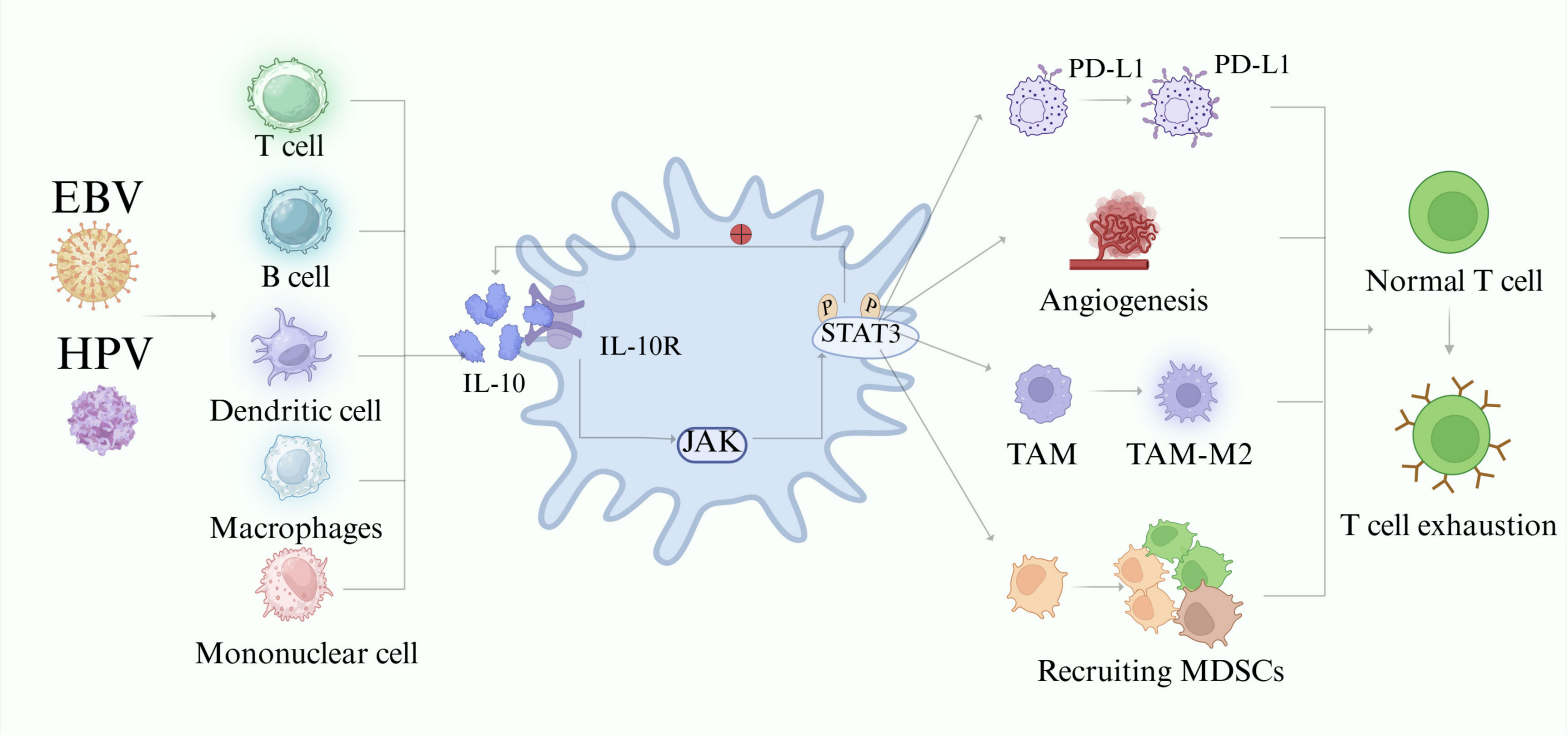
IL-10-mediated immunosuppression in the tumor microenvironment. Under viral stimulation, T cells, B cells, macrophages, DCs, and monocytes secrete IL-10. Upon binding to its receptor, IL-10 activates the JAK-STAT3 pathway. Activated STAT3 promotes increased IL-10 production, creating a positive feedback loop and driving multiple mechanisms that promote tumor progression. These include upregulating PD-L1 expression on tumor cells, promoting angiogenesis, inducing polarization of TAMs toward the M2 phenotype, and recruiting MDSCs. These actions lead to T-cell functional exhaustion of T cells. Created using BioRender.com.

Interleukin-10 (IL-10) is primarily known for its anti-inflammatory properties and plays a crucial role in preventing excessive immune activation and maintaining immune homeostasis. In an immunohistochemical analysis of 133 laryngeal carcinoma specimens, IL-10 was identified as a prognostic risk factor for laryngeal cancer (Sun et al., 2018). Coinfection with HPV and EBV could synergistically amplify the pro-neoplastic effects of IL-10 (**Figure 4**). Single HPV and EBV infections, as well as coinfection with both viruses, promote IL-10 secretion by specific subsets of regulatory T cells, B cells, macrophages, monocytes, dendritic cells (DCs), and T helper cells (Chen et al., 2024). IL-10 binds to its receptor (IL-10R) to form a heterodimer (Berti et al., 2017). This activation stimulates the Janus kinase/signal transducer and activator of transcription 3 (JAK-STAT3) signaling cascade, and the activated STAT3 pathway further promotes IL-10 secretion through positive feedback (Huynh et al., 2017). Phosphorylated STAT3 recruits and activates myeloid-derived suppressor cells (MDSCs), which suppress T-cell responses. Additionally, it induces VEGF production and promotes tumor angiogenesis. Upon activation, JAK-STAT3 translocates to the nucleus and mediates the transcription of IL-10-immunosuppressive target genes. Furthermore, it enhances PD-L1 expression on tumor cell surfaces. This weakens T-cell function and facilitates tumor cell escape from immune surveillance via interactions with PD-1 on the T cells. The EBV BCRF1-encoded viral IL-10 (vIL-10) is a homologue of IL-10 (Stewart and Rooney, 1992). In animal studies, enhanced expression of vIL-10 in mice increased acute pathogenicity. However, it does not affect viral latency or reactivation during acute infection, and it plays a specific role in expanding the permissive host cell population, promoting the initial survival and spread of the virus within the infected cells (Lindquester et al., 2014). Furthermore, vIL-10 suppresses co-stimulatory molecules, uses gp42 to block cross-presentation, and disrupts the IFN signaling pathway via BZLF1, thereby promoting immune evasion (Salek-Ardakani et al., 2002; Jochum et al., 2012; Gujer et al., 2019).

Additionally, in the presence of IL-10, tumor-associated macrophages (TAMs) have been observed to shift to the M2 phenotype in HPV- and EBV-infected cells (Shivarudrappa et al., 2024; Chen et al., 2024; Xiang et al., 2024). TAM-M2 cells reduce the release of pro-inflammatory cytokines and growth factors, thereby providing an immunosuppressive environment conducive to tumor progression (Chen et al., 2019, 2024). Within the tumor microenvironment (TME), IL-10 also suppresses antigen-presenting cell maturation and activity, limits the ability of DCs to present tumor antigens, and impedes adaptive immune responses to facilitate immune evasion (Rallis et al., 2021; Mirlekar, 2022), reducing the production of pro-inflammatory cytokines such as IFN-γ and TNF-α (Bruchhage et al., 2018). IL-10 weakens the effector functions of cytotoxic T lymphocytes. Conversely, the immunosuppressive environment promotes IL-10 secretion, limiting its ability to control tumor growth (Groux et al., 1998; Li et al., 2020).

In adaptive immune evasion, mechanisms documented in viral oncology reveal that both mechanisms may synergistically reduce antigen presentation: HR-HPV inhibits human leukocyte antigen class I (HLA-I) transport to the Golgi apparatus via E5 and suppresses the major histocompatibility complex class I (MHC-I) heavy-chain promoter activity through E7, thereby inhibiting gene transcription, lowering HLA-I expression levels, and diminishing CD8^+^ T-cell responses (Georgopoulos et al., 2000; Jemon et al., 2016; de Freitas et al., 2017). The EBV nucleoprotein EBNA1 inhibits MHC-I-derived antigen peptide presentation (Levitskaya et al., 1995). The BGLF5 gene product, initially identified as an alkaline nucleic acid exonuclease, suppresses *de novo* protein synthesis, leading to loss of cell surface HLA-I molecules (Rowe et al., 2007). BNLF2a blocks the transporter associated with antigen processing (TAP) peptide transport (Horst et al., 2009). BILF1 may downregulate MHC-I molecules by enhancing endocytic degradation, thereby weakening CD8^+^ T-cell responses (Gujer et al., 2019; Singh et al., 2025). EBV disrupts CD4^+^ T-cell activation by impeding MHC-II/peptide-T-cell receptor (TCR) interactions via the gp42/gH/gL complex (Ressing et al., 2003). Cells coinfected with HPV and EBV could, in theory, synergistically create a low MHC-I and MHC-II environment for tumor cells, suppressing the detection of cells harboring latent and replicating viruses by CD8^+^ and CD4^+^ T lymphocytes.

### 2.6 Integrative perspective: the functional sequence of the “two-hit” model

The multifaceted synergy between HPV and EBV, as detailed in the preceding sections, is underpinned by a critical functional sequence that defines the proposed “two-hit” model. It is crucial to emphasize that the latent infection state of EBV in our model is a specific adaptation to the aberrant and differentiation-deficient epithelial microenvironment reshaped by preceding HPV infection. The first hit, mediated by HPV, extends beyond the canonical inactivation of p53 and pRb. Its fundamental role is to reprogram the epithelial niche, disrupting differentiation and innate immune sensing, thereby establishing a cellular microenvironment permissive to viral persistence. This altered microenvironment effectively facilitates the establishment of EBV latency, setting the stage for sustained coexistence (Makielski et al., 2016; Guidry et al., 2019). Within this preconditioned landscape, the second hit is executed by EBV. Latent proteins such as LMP1 engage in extensive molecular cross talk with persistent HPV oncoproteins, resulting in amplified oncogenic perturbations in DNA damage response, cellular metabolism, and immune evasion pathways (Shimabuku et al., 2014). Thus, the model emphasizes that HPV-led microenvironmental remodeling is a pivotal precursor event, enabling and potentiating the subsequent collaborative strike from EBV. While definitive proof of infection order and the exact sequence of molecular events in LSCC *in vivo* remains challenging, this functional logic, drawn from extrapolated data, provides a coherent framework for understanding the pronounced oncogenic synergy observed in coinfected lesions.

## 3 HPV and EBV coinfection and clinical translation

LSCC poses a significant threat to patients’ quality of life, and HPV status may serve as a prognostic factor for patients with advanced head and neck squamous cell carcinoma (HNSCC) (LSCC and OPSCC) (Ko et al., 2017; Bates et al., 2019). In HPV–EBV-coinfected colorectal cancer, the proportion of moderately/well-differentiated tumors was higher than in those with a single HPV or EBV infection (Gupta et al., 2020a). HPV–EBV coinfection acts as a cancer-promoting factor in prostate cancer (Nahand et al., 2021). Studies on HPV–EBV coinfection in cervical cancer suggest that EBV infection may be a poor prognostic factor (Cameron et al., 2020). Although studies on the impact of HPV–EBV coinfection on laryngeal cancer prognosis remain limited, findings from other cancers suggest that it influences prognosis. Consequently, improving the early diagnosis rate of HPV–EBV-coinfected laryngeal cancer is necessary. The diagnosis of HPV and EBV-coinfected laryngeal cancer requires the combined use of multiple biomarkers. At the histological level, HPV activity is typically initially screened by p16 immunohistochemistry and subsequently confirmed by molecular validation (e.g., polymerase chain reaction or fluorescence *in situ* hybridization) of viral DNA or RNA (Thomas et al., 2025). Concurrent testing for EBV DNA is employed to confirm EBV coinfection (Liu et al., 2021). This combined virological approach enhances the sensitivity of HPV–EBV coinfection detection in LSCC. Liquid biopsy technology, now mature for head and neck cancers with single HPV(e.g., OPSCC) or EBV (e.g., nasopharyngeal carcinoma) infection (Chera et al., 2019; Huang et al., 2019), may offer a promising non-invasive tool for diagnosing and assessing the prognosis of patients with HPV/EBV-coinfected laryngeal cancer. Furthermore, drawing on the successful experience of plasma EBV DNA screening for nasopharyngeal can improve the early diagnosis rate (Chan et al., 2017). Future strategies may establish enhanced management protocols for high-risk populations with coinfections in laryngeal cancer. Patients who are seropositive for HPV antibodies and dual positive for EBV Viral Capsid Antigen Immunoglobulin A and Immunoglobulin G (VCA-IgA/IgG) antibodies, concurrently exhibiting smoking habits or multiple sexual partners, can be enrolled in intensive surveillance programs to identify early-stage cancers.

Regarding prevention, tumor-associated antigens (TAAs) are promising targets for cancer vaccine design in various malignancies. TAAs are non-mutated endogenous proteins in cancer cells, such as Mucin 1 (MUC1) and carcinoembryonic antigen. MUC1 is a glycoprotein present on all epithelial cell surfaces, and its abnormal expression correlates with the cancerous phenotype, making it an ideal target for developing vaccines against epithelial cancers, a strategy not yet specific to LSCC (Wang et al., 2018; Cheng et al., 2021).

Drawing parallels from the broader field of oncometabolism, glycolytic pathway activation and lactate accumulation may act synergistically in HPV–EBV-coinfected LSCC. During glucose metabolism, upregulation of glycolysis and downregulation of mitochondrial oxidative phosphorylation can suppress T-cell function and reduce the efficacy of immune checkpoint inhibitors (ICIs) (Jia et al., 2021). Consequently, targeting glycolysis is hypothesized to counteract its role in promoting immune evasion (Mi et al., 2015; Strauss et al., 2018). Additionally, macrophage reprogramming has been observed, and TAMs predominantly convert to M2 type in the TME, exerting pro-tumorigenic effects and creating an immunosuppressive microenvironment. Metabolic reprogramming of TAMs or blocking of the colony-stimulating factor 1 (CSF-1)/IL-10 signaling pathway represents a potential strategy to reverse their immunosuppressive effects (Chen et al., 2022; Zhang et al., 2023).

HPV–EBV coinfection may amplify the synergistic pro-tumorigenic effects of the cGAS-STING pathway. Recent studies in various cancers indicate that the administration of various small molecules and nanoparticle-based STING agonists, such as ADU-S100 and c-di-GMP carriers, robustly induces type I IFN production and CD8^+^ T-cell infiltration in mouse tumor models, resulting in significant tumor growth inhibition (Sivick et al., 2018). Combination with radiotherapy/chemotherapy or anti-programmed cell death protein 1/programmed cell death ligand 1 (PD-1/PD-L1) antibodies produces synergistic antitumor effects and prolongs progression-free survival in preclinical settings (Meric-Bernstam et al., 2023). However, early studies on ADU-S100 demonstrated low objective response rates (<10%), potentially attributable to species differences and challenges in payload stability and targeting associated with systemic drug delivery (Luke et al., 2023). This may also be because cGAS-STING inherently possesses anti-inflammatory and pro-tumorigenic effects *in vivo* due to the complexity of the TME, and its activation can also cause PD-L1 upregulation, reducing the efficacy of immunotherapy (Huang et al., 2025; Tufail et al., 2025). Tumor-specific STING agonists remain under investigation and may provide valuable insights into tumor-specific drug therapies in the future (Duvall et al., 2023).

Currently, emerging bispecific antibody therapies target immune evasion. Unlike conventional monoclonal antibodies, bispecific antibodies are engineered to recognize and bind to two distinct antigens. This enables them to link cancer cells with immune cells, thereby enhancing the immune response against tumors. A study indicated that the bispecific antibody M7824, which targets PD-L1 and TGF-β, demonstrated significant clinical efficacy in NSCLC when administered in combination with a PD-1 inhibitor (Lan et al., 2018; Strauss et al., 2018). Next-generation PD-L1-targeting bispecific antibodies combined with other immune checkpoints have demonstrated significant antitumor activity in preclinical and early clinical studies (Cheng et al., 2024; Li et al., 2024). Coinfection in LSCC causes PD-L1 upregulation on tumor cell surfaces and TGF-β accumulation, suggesting that these novel therapies could potentially enhance the efficacy of existing ICIs for laryngeal cancer.

Finally, artificial intelligence (AI) can participate in every aspect of the HNSCC process, from early detection and diagnosis to treatment planning, identifying mutations through genomic data analysis, developing targeted therapies, and completing the monitoring and surveillance of tumor cells (Pham et al., 2024). Gene editing technologies, such as Clustered Regularly Interspaced Short Palindromic Repeats/CRISPR-associated protein 9 (CRISPR/Cas9), can be employed to modify immune and cancer cells within the TME, thereby enhancing the efficacy of immunotherapy. This approach has been investigated in numerous cancer types, including HNSCC (Feng et al., 2024). Future research should further explore the role of AI and gene editing in LSCC management and the effective integration of these technologies into clinical practice (Jayawickrama et al., 2024; Chiesa-Estomba et al., 2025).

## 4 Summary and outlook

The complex pathogenesis of LSCC provides a compelling framework for understanding the functional “two-hit” model of HPV and EBV coinfection. HPV initiates epithelial differentiation disruption and activates carcinogenic pathways via E6/E7 oncoproteins, while EBV synergistically suppresses DDR through the LMP1 protein, promoting malignant proliferation and resistance to apoptosis. The two viruses also interact to generate a cascading effect: HPV downregulates the transcription factors required for EBV lysis, forcing EBV to maintain latent infection and increase genomic instability. Concurrently, they synergistically elevate TGF-β levels to drive the EMT and metastatic potential. At the metabolic level, HPV–EBV coinfection enhances aerobic glycolysis via pathways, including GLUT1/GLUT4 upregulation, HIF-1α stabilization, and excessive LDH-A activation. This causes lactate accumulation in the microenvironment, promoting angiogenesis and shaping an immunosuppressive microenvironment. Immune evasion is posited as a core carcinogenic mechanism of coinfection. Viruses synergistically suppress TLR signaling and abnormally activate cGAS-STING pathways, while inducing IL-10 overexpression to weaken innate immunity. At the adaptive immune level, they inhibit CD8^+^ T-cell responses by downregulating HLA-I molecule expression, forming a dual immune barrier. While the above synergistic mechanisms are plausible extrapolations, clinical studies have indicated that HPV–EBV-coinfected LSCCs themselves predominantly exhibit a moderately or highly differentiated phenotype, potentially correlating with a poor prognosis. To move from extrapolation to LSCC-specific validation, future research should prioritize direct experimental verification of this functional sequence. Particular focus should be placed on quantifying how coinfection lowers the oncogenic threshold and accelerates tumor progression compared to single-virus infection. The development of temporally regulated *in vitro* and *in vivo* coinfection models will be critical to corroborate the causal contribution of HPV-mediated preconditioning and to identify the critical molecular events at each sequential stage. It is highly recommended that future work employ high-dimensional technologies—such as single-cell RNA sequencing, spatial transcriptomics, and multiplex immunofluorescence—to systematically profile and compare the tumor immune microenvironments across HPV-monoinfected, EBV-monoinfected, HPV/EBV-coinfected, and virus-negative LSCC patients. These investigations will be pivotal in determining whether the two viruses merely coexist or functionally collaborate to establish a uniquely immunosuppressive niche, thereby accelerating malignant progression. Translating these mechanistic insights, multi-viral biomarker panels and pathogen-targeted combination therapies may optimize the clinical management of HPV–EBV-coinfected LSCC. Current prevention and treatment strategies are diversifying. HPV vaccines can prevent laryngeal cancer and other cancers originating from epithelial cells. TAA cancer vaccine development is a future priority, and emerging approaches, such as glycolytic pathway inhibitors, ICIs, STING agonists, and bispecific antibody immunotherapies, offer precise treatment directions. However, overcoming the constraints of complex TME is essential. With AI technologies now integrated into TME analysis, breakthrough optimizations in preventing and treating the system for coinfected laryngeal cancer are anticipated.
